# Not so natural: hidden NSAID in traditional medicine causing granulomatous interstitial nephritis

**DOI:** 10.1093/ckj/sfag212

**Published:** 2026-06-23

**Authors:** Philippe Le Moal, Jean-Claude Alvarez, Pamela Duguès, Romain Batton, Charlotte Mussini, Pierre Galichon

**Affiliations:** Nephrology Department, CHU Ambroise Paré (AP-HP), Boulogne Billancourt, France; UVSQ-Paris Saclay University, Montigny-le-Bretonneux, France; Department of Pharmacology and Toxicology, Raymond Poincaré Hospital, MONITOR Platform, GHU AP-HP. Paris-Saclay, UVSQ/Paris-Saclay University, Inserm U-1018, CESP (Center for Research in Epidemiology and Population Health), Team MOODS, Garches, France; Department of Pharmacology and Toxicology, Raymond Poincaré Hospital, MONITOR Platform, GHU AP-HP. Paris-Saclay, UVSQ/Paris-Saclay University, Inserm U-1018, CESP (Center for Research in Epidemiology and Population Health), Team MOODS, Garches, France; UVSQ-Paris Saclay University, Montigny-le-Bretonneux, France; Medical and Surgical Intensive Care Unit, CHU Ambroise Paré (AP-HP), Boulogne Billancourt, France; UVSQ-Paris Saclay University, Montigny-le-Bretonneux, France; Pathology Department, CHU Kremlin Bicêtre (AP-HP), le Kremlin-Bicêtre, France; Nephrology Department, CHU Ambroise Paré (AP-HP), Boulogne Billancourt, France; UVSQ-Paris Saclay University, Montigny-le-Bretonneux, France

**Keywords:** acute interstitial nephritis, hidden pharmaceuticals, high-resolution mass spectrometry, traditional medicine

## Abstract

Acute interstitial nephritis (AIN) is a common finding on kidney biopsies indicated for acute kidney injury (AKI). Histologically, it translates into an interstitial inflammatory infiltrate. In less than 1% of biopsy-confirmed nephropathies, this interstitial nephritis presents as granulomas form [1]. AIN can result from multiple causes, including autoimmune diseases, infections, and drug side effects. Here, we report an original case illustrating the importance of a tenacious etiological investigation. A 39-year-old patient using traditional Sri Lankan medicine required intensive care for severe AKI with the need for dialysis, associated with other visceral and skin manifestations. Kidney biopsy revealed AIN with many eosinophilic neutrophils and perivascular granulomas, which were suspected to be allergic in origin. His recovery with corticosteroid therapy enabled us to stop dialysis and achieve full renal recovery. We decided to analyze these traditional medicines that the patient provided and of unknown composition. To this end, we used high-resolution mass spectrometry, which enabled us to identify many chemicals known in Western societies to be nephrotoxic and which could lead to severe allergies.

## THE CASE

A 39-year-old Pakistani man took Sri Lankan traditional medicine for ∼2 weeks to treat neck pain (Fig. 2).

Following this, he developed digestive problems, including diarrhea and vomiting, accompanied by a general feeling of discomfort. This was followed by the appearance of an extensive maculopapular rash affecting ∼30% of his body surface area. These symptoms prompted him to visit emergency department.

Initial laboratory tests revealed severe anuric acute kidney injury (AKI) with a creatinine level of 548 µmol/L, associated with proteinuria of 3 g/g creatinine, microscopic hematuria, and frank leukocyturia without infectious pathogen identified on urine residues. Moderate hepatic cytolysis, at approximately twice the upper normal level, was also noted, as well as marked peripheral hyper eosinophilia at 1.6 G/L. Anti-nuclear antibody (ANA), anti-neutrophil cytoplasmic antibody (ANCA) , and anti-glomerular basement membrane antibody (GBM) were negative.

An abdominal-pelvic CT scan revealed no notable morphological abnormalities; the kidneys were normal in size (∼11 cm long) and there was no dilation of the pyelocaliceal cavities.

Due to the severity of the renal failure and persistent anuria, the patient required three sessions of renal replacement therapy in the intensive care unit, after which he was transferred to the nephrology department for an etiological investigation.

The patient and his family told us that the only treatment taken before the onset of symptoms was the traditional medicine described above.

**Figure 1:**
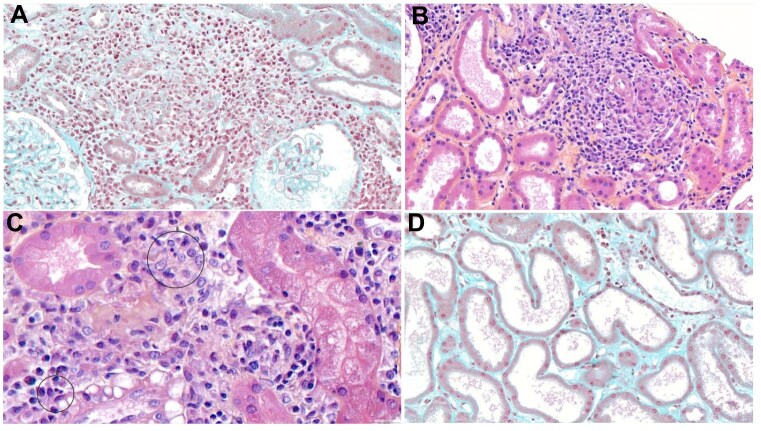
Kidney biopsy. (A and B) Interstitial nephritis with perivascular granulomas. (C) Presence of eosinophil polynuclear cells in the interstitium. (D) Acute tubular necrosis.

A renal biopsy was performed. Pathological examination revealed significant acute tubular necrosis associated with acute tubulointerstitial nephritis and a lymphoplasmacytic infiltrate containing some eosinophils. Two perivascular interstitial granulomas were identified without giant cells or necrosis, which suggests a drug-induced origin. The interstitium was the site of significant edema. The glomeruli appeared optically normal. Immunofluorescence was negative (Fig. 1).

Corticosteroid therapy was initiated with three intravenous pulses of methylprednisolone, followed by oral prednisone at a dosage of 1 mg/kg/day and then a gradual taper.

During treatment, gradual recovery of diuresis and kidney function was observed. Last creatinine one month later was 68 µmol/L. Additionally, the clinical presentation of the skin and digestive system, as well as inflammatory biological parameters, normalized rapidly after the introduction of corticosteroid therapy.

**Figure 2:**
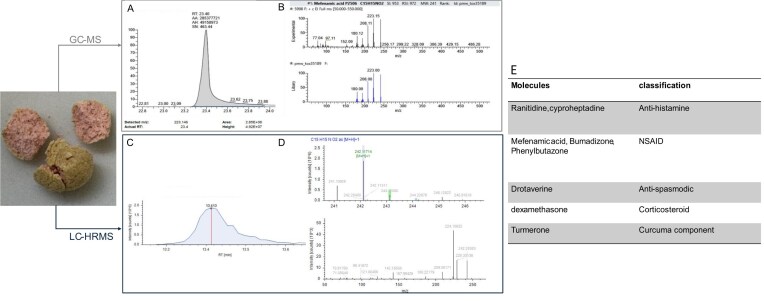
Example of mass spectrometry analysis of the main compound in one of the traditional medicine products: (A) GC–MS chromatogram, with time on the *x*-axis and signal intensity on the *y*-axis. The peak centered at 23.40 min indicates that a compound elutes from the GC column at 23.40 min. (B) Top: Spectrum of the compound eluting at 23.40 min, with each vertical bar representing an ion fragment; bottom: comparison with the reference spectrum of the molecule proposed by the library (mefenamic acid). (C) LC–HRMS chromatogram. The peak centered at 13.413 min indicates that a compound elutes from the LC column at 13.413 min. (D) Top: The HRMS MS1 spectrum of the compound eluting at RT 13.413 min, providing the exact neutral molecular mass [M + H]+ of the compound; bottom: the HRMS MS2 spectrum of the compound eluting at RT 13.413 min, enabling structural identification and comparison with a collection of reference MS2 spectra associated with identified molecules (here mefenamic acid). (E) Composition of all compound identified in high-resolution mass spectrometry. GC–MS: gas chromatography–mass spectrometry; LC–HRMS: liquid chromatography–high-resolution mass spectrometry; RT: retention time.

We had these treatments analyzed by liquid chromatography coupled with high-resolution mass spectrometry (LC-HRMS) [2]. (example in Fig. 2A–D). The conclusions of the mass spectrometry are listed in Fig. 2E. There were multiple pain killers with NSAID. These unsaid NSAID are the most likely guilt in this acute interstitial nephritis (AIN). Patient confirmed the absence of any treatment beside herbal medicine.

## DESCRIPTION OF THE MASS SPECTROMETRY PROTOCOL USED TO IDENTIFY THE CHEMICAL COMPONENTS IN TRADITIONAL MEDICINE

Samples were weighed and ground into a fine powder. Ten milligrams of powders were solubilized in MeOH to obtain a concentration of 1 mg/mL. The solutions were sonicated for 10 min and centrifuged for 10 min. Fifty microliters of internal standard working solution were added to all samples prior to liquid chromatography high-resolution mass spectrometry (LC–HRMS) analysis. Liquid chromatography was performed on a Thermo Ultimate 3000 (Thermofisher, Les Ulis, France) pump and separation was carried out on a Hypersil GOLD column (100 × 2.1 mm × 1.9 µm, Thermo, USA) maintained at 40°C. Compounds were detected using an Orbitrap mass spectrometer (Q-Exactive, Thermo, USA). Data were acquired in data dependent acquisition mode according to a previously published method [3]. The masses of precursors and their related fragments ions were measured with a resolution of 70 000 and 17 500 FWHM at *m*/*z* 200, respectively, in the range 125–650 *m*/*z*. The mass isolation window was 1 *m*/*z*. The normalized collision energy (NCE) was set at 55%. Chromatographic data acquisition was performed using Xcalibur software (v.4.0, Thermo, USA). Raw data were processed with Compound Discoverer 2.0 software (Thermo, USA). Exact mass spectra identification was performed with the mzCloud™ data base (https://www.mzcloud.org/).

## DISCUSSION

Drug-induced AIN represents more than 50% of biopsy proven AIN [4]. There are many molecules involved, widely used in Western society, led by proton pump inhibitors, antibiotics, non-steroidal anti-inflammatory drugs, but also certain anti-epileptics and now checkpoint inhibitors in oncology [5, 6].

Granulomatous interstitial nephritis is a rare form of AIN. Drug-induced causes are well described in the literature and account for nearly half of all cases in some series [7], with the remainder being systemic granulomatous diseases such as sarcoidosis and, more rarely, IgG4-related disease, tuberculosis, and ANCA-associated vasculitis.

Treatment consists of discontinuing the molecule in question (and therefore identifying it) and, in cases of severe symptoms and in the absence of recovery despite discontinuing exposure to the causative agent, corticosteroids have been reported to be effective [8]. However, the difference in efficacy related to the mode of administration, whether oral or parenteral, with or without pulses, remains uncertain [9].

The emergence of new nephrotoxic molecules causing AIN, possibly in a granulomatous form, is likely to increase. Traditional medicines are among these and should be investigated in cases of AKI and/or histologically proven AIN. Some are already known to be responsible, such as Chinese herbs [10], but there are also other less well-known therapies that need to be documented in order to prevent the onset of their toxicity [11].

Traditional medicines may be gaining ground in Western society and in the era of globalized healthcare. It is important to keep in mind that these same traditional medicines remain receptive to modern medicine, increasing the risk of unintentional rechallenge. In our case, the presence of NSAIDs in the medications used by the patient led to the definitive contraindication of NSAIDs and ranitidine, non-essential medications with previously reported causality.

The expected impact of this clinical situation is not the novelty of the described disease or the technology used to reach the final diagnosis, but rather to alert clinicians that allergy diagnoses, which can have severe consequences and are likely to be underdiagnosed, can be clarified using accessible techniques such as mass spectrometry.

Furthermore, physicians should be aware that traditional medicines may contain chemical components such as painkillers or antibiotics. Identifying the causal component using high-resolution mass spectrometry as soon as possible may prevent relapse in the future, which could manifest in the same way, but with the ‘conventional’ use of these molecules.

## PATIENT CONSENT

The patient described in this report provided written informed consent for the publication of the clinical details and accompanying data. All identifying information has been removed to protect patient confidentiality.
